# Nature and perceived benefits of patient-initiated consultations in community pharmacies: A population survey

**DOI:** 10.1016/j.rcsop.2022.100194

**Published:** 2022-10-12

**Authors:** Guy Paré, Aude Motulsky, Alexandre Castonguay, Stéphanie Boulenger

**Affiliations:** a Research Chair in Digital Health, HEC Montréal, Montréal, Canada; b Research Center, Centre hospitalier de l’Université de Montréal, Montréal, Canada; Department of Management, Evaluation and Health Policy, School of Public Health, Université de Montréal, Montréal, Canada; c Faculté des sciences infirmières, Pavillon Marguerite-d'Youville, C.P. 6128 succ. Centre-ville, Montréal (Québec) H3C 3J7, Canada; d Center for Interuniversity Research and Analysis of Organizations, Montréal, Canada

**Keywords:** Community pharmacy, Counselling, Patient satisfaction, Primary health care, Surveys and questionnaires

## Abstract

**Background:**

The role of community pharmacists in enhancing patient care has received increased attention. However, there is a paucity of literature on the nature, frequency, and perceived impacts of patient-initiated consultations in community pharmacies.

**Objectives:**

We aim to describe the profile of patients seeking advice from community pharmacists as well as the nature and impact of those consultations.

**Methods:**

A survey was conducted with Quebec adults who had consulted a pharmacist in the previous four weeks. Data was collected in 2017 and 1104 agreed to participate (25.3%). Of those, 93 were withdrawn due to incomplete data and 98 failed to meet the inclusion criteria. Sample representativeness was ensured by quota sampling (gender, age) after stratification by region.

**Results:**

Among the 913 respondents, 46% had consulted a pharmacist more than once during the four weeks prior to the survey. Individuals with a university degree consulted less often than those without (1.97 vs. 2.17 times; *t* = 2.0; *p* < .05) and participants with one or several chronic diseases consulted more frequently than those having no chronic disease (2.18 vs. 1.94 times; *t* = 5.7; *p* < .05). Older adults (55+) consulted more often for themselves compared to younger (18–34) and middle-aged (35–54) adults (1.53 vs. 1.31 vs. 1.44 times; *F* = 4.0; *p* < .05). Concerning the consultations, 58% were related to medications and 33% to health problems. In terms of impacts, 81% of consultations were perceived to have prevented the use of other healthcare resources. Patient satisfaction with their consultations was high with an average score of 8.75 on a 10-point scale (SD = 1.63).

**Conclusions:**

Findings reveal that the reasons for consulting a community pharmacist are diverse, most being related to medications or health issues. Patients reported that pharmacists were able to manage most consultations without referring them to other health care resources or professionals, and their satisfaction with their consultation was high.

**MeSH terms:**

Community pharmacy; counselling; patient satisfaction; primary health care; surveys and questionnaires.

## 1 Introduction

Community pharmacies in several countries positively impact patient care because of their convenience as supported by the frequency of access by patients.^1^ In Canada, more than half of the adult population visits a community pharmacy once a week and sees a community pharmacist up to ten times more frequently than their family physician.^2^, ^3^, ^4^ Community pharmacists have long been viewed as a distributor of medicines, but this role is rapidly changing with the general public now seeing pharmacists as primary care providers.^5^ Community pharmacists are steadily rated as one of the most trusted health care professionals and over 8 out of 10 Canadian adults agree that allowing them to do more for patients will improve health outcomes.^3^

From a global perspective, the role of the community pharmacist in enhancing patient care has received increased attention.^6^, ^7^, ^8^ For instance, the International Pharmaceutical Federation (FIP) has recognized the important role played in primary care by community pharmacists, one that goes well beyond the distribution and dispensing of prescribed medications.^9^ In Canada, the will to expand and transform this role as a more efficient and patient-focused delivery of services is strongly envisioned.^10^ Indeed, community pharmacists are more than ever valued as primary care providers, as can be seen in the scope of their responsibilities.^11^ In the province of Quebec, where the present study was conducted, a considerable expansion of community pharmacists' rights and responsibilities occurred in 2015 and again in 2021 with the coming into force of Bills 41 and 31.12., 13., 14. In the process, community pharmacists were authorized to prescribe over-the-counter (OTC) drugs (i.e. non-prescription medicines) as well as certain laboratory tests and vaccines, to extend or adjust prescriptions, and even to administer certain vaccines and medications. Recognizing the importance of the work performed by pharmacists in improving primary care, in 2021 the Quebec government made some community pharmacy prescriptions and prescription adjustment services free for patients.^15^

While the types of patient care services that community pharmacists are providing vary considerably by country or even by provincial or state jurisdiction,^1^ patient counselling is recognized as one of the most important services delivered by these professionals.^16^, ^17^, ^18^ While there is no accepted definition of patient counselling, with the terms counselling, education, information provision, and communication being used interchangeably,^18^^,^^19^ there is substantial evidence that through these activities, pharmacists can identify and resolve drug-related problems,^20^^,^^21^ empower patients to adopt self-management behaviors,^22^ and optimize quality of care.^23^

While prior research has mainly investigated pharmacist-led counselling activities, the present study focuses on patient-initiated consultations. Recent studies have documented the variety of reasons for consulting in community pharmacies. These motives go well beyond medication management and range from advice on breastfeeding^24^ to support for end-of-life palliative care,^25^ monitoring chronic diseases,^26^ patient education about contraception,^27^^,^^28^ and assisting in healthy weight management.^29^ Other studies have shown that the advice and recommendations provided by community pharmacists are generally highly appreciated by patients.^30^, ^31^, ^32^

Despite these important findings, little is known about the nature, frequency, and perceived effects of patient-initiated visits to community pharmacies. To our knowledge, the observational study by Motulsky et al.^33^ is the only one which addressed these issues. In that study, pharmacists working in 11 community pharmacies in Quebec were asked to compile in a mobile application their patient-initiated consultations for a 4-week period. A sample of patients who initiated these consultations were recruited in pharmacies and agreed to participate in a first on-site interview and a follow-up interview by phone seven days later. Findings reveal that 1) community pharmacists provide approximately 18 patient-initiated consultations per work shift; 2) the level of patient satisfaction with these consultations is very high; and 3) 77% of the patients reported that their consultations with community pharmacists spared them a visit to their family physician's office or an emergency room.

The present study was conducted in parallel to the work reported in Motulsky et al.^33^ and performed by the same research team. While our previous results were based on data from a convenience sample of patients, in the present article we analyse data from a large representative sample of adults. Precisely, we aim to characterize the profile of patients seeking advice from community pharmacists as well as the nature of these consultations, assess the perceived effects of these consultations on patients' health status and consumption of health services, and determine the level of patient satisfaction with these consultations.

## 2 Methods

### 2.1 Research methodology and survey instrument

To achieve our objectives, a population-based survey was conducted using a sample of Quebec adults who could speak English or French and who had sought the advice of a community pharmacist in the previous four weeks, either for themselves or someone else (e.g., child, father, sister, aunt). The consultation with the pharmacist could have been in person at the pharmacy or by telephone. A series of questions on the nature, frequency, and perceived impacts of patient-initiated consultations were developed for the purpose of this study. The initial version of the survey instrument was pretested with a convenient sample of 10 adults (6 French speaking and 4 English speaking) who had recently consulted a community pharmacist. We conducted individual interviews during which we asked respondents to think aloud as they answered survey questions. We wanted to ensure that respondents interpreted and answered questions in the way in which our research intended. A few minor changes were made to the survey instrument based on the pretest. The final version of the questionnaire is available in Appendix A.

### 2.2 Data collection

Data was collected by the largest Canadian-owned market research company, Léger. The respondents were randomly selected from the firm's Web panel. In October 2017, the firm sent an email invitation to 4369 individuals who met the inclusion criteria described above. The email contained a secure, personalized Web link to the questionnaire. Each link was active for a specific period and was unique to each respondent, so it could only be used once (based on IP check). The survey was voluntary and no incentives (eg, monetary, prizes) were offered to participants. Respondents were able to review and change their answers (through a Back button) before completing the survey.

### 2.3 Data analysis

Both qualitative and quantitative analyses were conducted. First, we content analyzed the reasons mentioned by participants for consulting a community pharmacist (open-ended question). As suggested by Hsieh and Shannon,^34^ conventional content analysis was used given the descriptive and inductive nature of our study. Hence, we avoided using preconceived categories, instead allowing the categories to emerge from the data. Descriptive data analysis was performed to determine the profile of the participants and the nature of the consultations. Mean comparison testing (*t*-test and ANOVA) was conducted to determine the profile of patients who consult pharmacist most often as well as variations in level of satisfaction across respondents. Last, Chi-squared testing allowed us to examine pharmacists' recommendations per main reason for consultation. All statistical analyses were performed using the IBM SPSS software v28. The significance level for this study was 0.05.

### 2.4 Ethics approvals

All study procedures were approved by the HEC Montréal's research ethics committee on August 14, 2017 (#2018–2852).

## 3 Results

### 3.1 Profile of respondents

Of all the Léger's panel members contacted, 1104 agreed to participate in the survey, representing a response rate of 25.3%. Of this number, 93 responses had to be withdrawn due to incomplete data (completion rate of 90.8%). In addition, another 98 questionnaires were removed from the sample because the only stated reason for the consultation concerned the refilling of an existing prescription (exclusion criterion). The final sample therefore consisted of 913 respondents.

Representativeness to the general adult population in Quebec was ensured by quota sampling (gender, age) after stratification by region. The maximum margin of error associated with the sample is estimated at 3.3%, 19 times out of 20. The results were weighted according to the following variables: gender, age, region, mother tongue, level of education and the presence of children in the home. Table 1 presents the profile of the respondents.

**Table 1:** Profile of the respondents (*N* = 913).

		Number of respondents	%
Sex	Male	428	47
	Female	485	53
Age group	18–24 years	76	8
	25–34 years	157	17
	35–44 years	145	16
	45–54 years	159	17
	55–64 years	159	17
	65 years and older	217	24
Mother tongue	French	706	77
	English	139	15
	Other	68	7
Highest completed level of education	Primary	5	<1
	Secondary	343	38
	College	257	28
	University	298	33
	Prefer not to answer	10	1
Number of people in the household	1	203	22
	2	366	40
	3	145	16
	4	122	13
	5	58	6
	6 or more	15	2
	Prefer not to answer	4	<1
Gross family income	<20 K	93	10
	20 K – 39.9 K	166	18
	40K – 59.9 K	174	19
	60 K – 79.9 K	141	15
	80 K – 99.9 K	95	10
	100 K or more	125	14
	Prefer not to answer	119	13
Family physician	Yes	776	85
	No	137	15
Number of consultations with a pharmacist in the previous four weeks	1	495	54
	2	202	22
	3	101	11
	4	52	6
	5	18	2
	6 or more	45	5
Chronic illness (in the person concerned by the consultation in a pharmacy)	No diagnosis	449	49
	One diagnosis	282	31
	More than one diagnosis	182	20

In the four weeks prior to data collection, our respondents had consulted a community pharmacist 2.1 times (SD = 2.0), on average. Overall, one quarter of the respondents (24%) had consulted a pharmacist three or more times. The results presented in Table 2 suggest that participants aged 18–34 years consulted pharmacists most often, whereas those aged 55 years and older consulted least often (*p* < .05). The groups that appear to consult more frequently for themselves are people aged 55+ and people with chronic illness(es). For their part, the groups that appear to consult more for a child are women and young adults (18–34 years). In terms of level of education, we divided our sample into two subgroups: those with a university degree and those without. Our results show that people without a university degree consult a community pharmacist more often than those with a university education (*p* < .05). We also found that adults with one or more chronic diseases consult a community pharmacist more than those without such illnesses (p < .05). Lastly, having a family physician does not appear to influence the frequency with which individuals consult community pharmacists.

**Table 2:** Determinants of seeking out a community pharmacist (*N* = 913).

		N	Number of patient-initiated consultations				*t* or *F* test
			For themselves	For a child	For a relative	Total	
Sex	Male	428	1.37	0.26	0.34	1.97	*t* = 3.6; ns
	Female	485	1.51	0.38	0.31	2.20	
Age	18–34 years	233	1.31	0.64	0.38	2.33	*F* = 4.0; p < .05
	35–54 years	304	1.44	0.44	0.30	2.18	
	55 years or +	376	1.53	0.04	0.30	1.87	
University degree (*n* = 903)	No	654	1.51	0.32	0.35	2.17	*t* = 2.0; p < .05
	Yes	249	1.28	0.34	0.25	1.86	
Chronic illness(es)	No	449	1.19	0.47	0.28	1.94	*t* = 5.7; p < .05
	Yes	464	1.69	0.19	0.36	2.18	
Family physician	Yes	776	1.46	0.34	0.32	2.12	*t* = 1.1; ns
	No	137	1.35	0.25	0.33	1.92	

ns = non-significant test.

As mentioned above, we asked participants to answer a series of questions related to a single consultation, that is, the one they considered the most important. First, Table 3 shows that these consultations were primarily for information or advice related to one or more medications (either prescribed or not), health issues or problems and, to a much lesser extent, other types of general advice (e.g., pregnancy tests, natural products, insurance). Appendix B presents a list of the most common reasons under each category. Table 3 also indicates that most of the consultations sought information for the respondents themselves. Last, a significant share of the respondents had attempted to consult another health professional (primarily a family physician) prior to visiting the pharmacy.

**Table 3:** Nature of the community pharmacy consultations (*N* = 913).

		Number of respondents	%
Main reason for the consultation	Medications	528	58
	Health problems	297	33
	Other reasons	77	8
	Prefer not to answer	11	1
For whom?	Oneself	736	81
	Someone else	177	19
Did you try to consult another professional beforehand?	Yes	199	22
	No	714	78
If yes, which kind? (*n* = 199)	Family physician	94	47
	Physician or nurse in a walk-in clinic	41	21
	Emergency physician	25	13
	Medical specialist	13	6
	Health information line (nurse)	14	7
	Other	12	6
If yes, were you able to reach this professional? (n = 199)	Yes	171	86
	No	28	14

Next, we examined the nature of the advice given by community pharmacists. Table 4 reveals that in 16% of the patient-initiated consultations, the pharmacist recommended a visit to the family physician. Pharmacists recommended going to a walk-in clinic or to the emergency room in 5% and 3% of all consultations, respectively. These three recommendations were more frequent when patients came to the pharmacy to discuss a health problem than when the primary reason for the visit was medication-related. Lastly, pharmacists recommended to make an appointment with a specialist physician (3% of all consultations) or call the 811-info line (<1%).

**Table 4:** Pharmacists' recommendations during patient-initiated consultations (*N* = 902).

	All consultations (*n* = 902)		By type of main reason for the consultation						
			Medication (*n* = 528)		Health issue or problem (*n* = 297)		Other reasons (*n* = 77)		Chi-squared test
	n	%	n	%	n	%	n	%	
Make an appointment with a family physician	145	16	73	14	63	21	9	12	p < .05
Go to a walk-in clinic	43	5	9	2	34	11	0	0	*p* < .001
Go to the emergency room	26	3	8	2	15	5	3	4	p < .05
Make an appointment with a medical specialist	23	3	12	2	8	3	3	4	ns
Call the health information 811 line	4	<1	1	<1	3	1	0	0	ns

ns = non-significant test.

The results in Table 5 indicate a wide range of perceived outcomes resulting from consulting a community pharmacist. First and foremost, 81% of the respondents reported that such consultations prevented the use of health care resources (visits to emergency rooms, visits to a walk-in clinic, appointments with family or specialist physician, calls to 811-info line). Precisely, 22% said that consultations with a pharmacist allowed them to avoid visiting a family physician. One out of five respondents said that they were able to avoid a visit to an emergency room or a walk-in clinic. These two types of visits were more likely to be avoided when the visit to the pharmacy was related to a health problem. To a lesser extent, these consultations prevented patients from calling the 811-info line (9%) or making an appointment with a specialist physician (6%).

**Table 5:** Perceived outcomes resulting from consulting a community pharmacist (*N* = 902).

*The consultation with the pharmacist prevented…*	All consultations (*n* = 902)		By type of main reason for the consultation						
			Medication (*n* = 528)		Health issue or problem (*n* = 297)		Other reasons (*n* = 77)		Chi-squared test
	n	%	n	%	n	%	n	%	
Making an appointment with a family physician	201	22	128	24	59	20	14	18	ns
Going to an emergency room	171	19	58	11	102	34	11	14	p < .001
Going to a walk-in clinic	171	19	82	16	75	25	14	18	*p* < .005
Calling the 811-info line	81	9	49	9	29	10	3	4	ns
Making an appointment with a specialist physician	51	6	26	5	19	6	6	8	ns
Making an appointment with another healthcare professional	53	6	25	5	20	7	8	10	ns
Having an improved quality of life	231	26	130	25	89	30	12	16	p < .05
Experiencing less anxiety	203	23	136	26	52	18	15	19	p < .05
Avoiding deterioration in a health condition	202	22	83	16	98	33	21	27	p < .001
Recovering faster	158	18	70	13	81	27	7	9	p < .001
Avoiding missing work or school	71	8	27	5	39	13	5	6	p < .001

ns = non-significant test.

Furthermore, patients felt that their consultation with the pharmacist had a positive effect on their perceived quality of life (26%), decreased their level of anxiety (23%), stabilized their health condition (22%), allowed a faster recovery (18%), and prevented them from missing work or school (8%). Apart from the effect on level of anxiety, all the other benefits related to health status seem to be more pronounced when the consultation concerned a health problem.

Table 6 shows that some of the abovementioned benefits seem to vary between participants. Indeed, our results first indicate that young adults (18–34) and, to a lesser extent, individuals with no university degree are the ones who most perceive having avoided a visit to the emergency department because of the advice or recommendation given by the pharmacist. Further, our findings reveal that older adults (55+) and, to a lesser extent, people with one or more chronic diseases, are those who perceive that they have avoided a visit to their family doctor the most thanks to the pharmacy consultation. Last, men perceived that they avoided a visit to another healthcare professional (e.g., dentist, chirotherapist) because of their consultation with a pharmacist more than women.

**Table 6:** Perceived effects by groups of patients.

		A visit to an emergency room (*n* = 171)		A visit to a walk-in clinic (*n* = 171)		A visit to the family physician (*n* = 201)		A visit to a specialist physician (*n* = 51)		A call to the 811-info line (*n* = 81)		A visit to another healthcare professional (*n* = 53)	
		n	%	n	%	N	%	n	%	n	%	n	%
Sex	Men	84	49	85	50	83	41	29	57	36	44	34	64
	Women	87	51	86	50	118	59	22	43	45	56	19	36
	Chi-square	ns		ns		ns		ns		ns		p < .05	
Age	18–34 years	65	38	46	27	37	18	14	27	21	26	17	32
	35–54 years	59	35	64	37	62	31	17	33	28	35	16	30
	55+ years	47	27	61	36	102	51	20	39	32	40	20	38
	Chi-square	p < .001		ns		p < .005		ns		ns		ns	
Chronic illness(es)	No	90	53	87	51	82	41	20	39	34	42	21	40
	Yes	81	47	84	49	119	59	31	61	47	58	32	60
	Chi-square	ns		ns		p < .05		ns		ns		ns	
University degree	No	134	78	127	74	148	74	41	80	53	65	37	70
	Yes	37	22	44	26	53	26	10	20	28	35	16	30
	Chi-square	p < .05		ns		ns		ns		ns		ns	

ns = non-significant test.

Finally, patient satisfaction with their consultations was 8.75 (average) on a 10-point scale (SD = 1.63) (see Q16 in Appendix A). The results in Table 7 indicate some variations in satisfaction depending on the nature of the consultation and the respondent profile. For instance, we note that the main reason for a consultation has an impact on patient satisfaction, as consultations concerning one or more medications appear to generate more satisfaction than those related to health problems. In terms of the sociodemographic profile, women seem slightly more satisfied than men with the advice provided by community pharmacists. Age is also positively correlated with level of satisfaction. Since age is associated with chronic disease, it is not surprising to find that respondents diagnosed with one or more chronic diseases have higher levels of satisfaction than those without. Furthermore, patients with a family physician were more satisfied with the advice provided by their community pharmacist than those without. Last, our results show that having a university degree had no impact on patient satisfaction.

**Table 7:** Patient satisfaction with their consultation with a community pharmacist (N = 913).

		Mean	Standard deviation	*t* or F test
Sex	Men	8.62	1.68	*t* = 2.4; p < .05
	Women	8.88	1.57	
Age	18–34	8.31	1.66	F = 22.0; p < .001
	35–54	8.61	1.76	
	55+	9.15	1.39	
University degree	No	8.79	1.59	*t* = 1.0; ns
	Yes	8.67	1.72	
Chronic illness(es)	No	8.56	1.72	*t* = 3.6; p < .001
	Yes	8.94	1.51	
Family physician	Yes	8.84	1.54	*t* = 3.8; p < .001
	No	8.28	1.99	
Main reason for consulting	Medication	8.91	1.48	*t* = 3.6; p < .001
	Health problem	8.50	1.74	

ns = non-significant test.

## 4 Discussion

### 4.1 Principle findings

While prior research has measured patient satisfaction with services delivered in community pharmacies,^35^^,^^36^ the present study focuses on one of the most important services performed by community pharmacists, namely, patient counselling. Precisely, our main objectives were to characterize the profile of individuals seeking advice from community pharmacists and the nature of the recommendations provided by pharmacists, identify the perceived effects of patient-initiated consultations, and assess patients' overall level of satisfaction with these consultations. To achieve our goals, a population-based survey was conducted using a sample of 913 adults in Quebec, Canada.

Findings first show that 46% of the respondents consulted a community pharmacist more than once in the four weeks preceding the survey; and while participants with more education consulted less, those with chronic diseases consulted more. The number of consultations for oneself tended to increase with patient age, which was expected given that consumption of medicines increases with age.^37^^,^^38^ However, while respondents aged 55 years and older tended to consult more for themselves, those aged 18–55 years consulted more often for a child compared to older respondents. Regarding the nature of the consultations themselves, 58% were related to medications, 33% to health problems, and 8% to other reasons (eg, probiotics, insurance). These findings are very similar to those reported in a recent study conducted in Portugal where 36% of the survey respondents (*N* = 1114) used the community pharmacy as a first resource when seeking to treat a minor ailment and 54% used it as a first resource when seeking answers about medicines.^5^

Next, our results provide insights into the role played by community pharmacists in primary health care. First, 22% of respondents reported that the pharmacist's advice or recommendation allowed them to avoid visiting their family physician while 19% said that they avoided a visit to an emergency room or a walk-in clinic. Second, 22% of respondents reported that they had tried to reach another health care professional before consulting the pharmacist, and 86% of them succeeded in reaching them and 14% did not. Third, approximately one-quarter of the consultations analyzed here resulted in a referral to another health care professional, be it a family doctor, an emergency doctor, or a nurse in a walk-in clinic. Last, 81% of the patients reported that their visit to a pharmacy helped them avoid seeing another health care professional. These findings appear to be consistent with those reported in Motulsky et al.,^33^ who found that pharmacists referred their patients to another health care professional 15% of the time, and that patients reported that their visit to a pharmacy saved them consulting with another health care professional 77% of the time.

Interestingly, when consulting for a health problem, 66% of the respondents believed that the pharmacist's recommendation allowed them to avoid a deterioration in their condition, or that it directly contributed to a faster return to a better quality of life. Through their interventions and advice, community pharmacists therefore can help stabilize and even improve the health status of the patients consulting them. These findings have also been echoed in previous studies. Indeed, the extant literature indicates beneficial effects of community pharmacy consultations for various health conditions, including chronic diseases in general,^26^ hypertension,^39^ and type 2 diabetes.^40^

Considering the abovementioned benefits, respondents were either globally satisfied or very satisfied with pharmacists' counselling services. These results are consistent with other studies conducted in Indonesia,^41^ Finland,^16^ and Portugal.^5^ High patient satisfaction may also be related to the fact that the general public tends to view community pharmacists not only as experts on drugs, but also as trustworthy health care professionals, just like physicians and nurses.^42^

### 4.2 Limitations and suggestions for future research

Certain methodological limitations need to be considered when interpreting the results of this study. First, the responses relied on self-reporting and included only those individuals who participated in the Web panel managed by the market company. Second, given the low participation rate and the fact that our sample consists of patients in a single Canadian province, our findings may not be generalizable to other jurisdictions and do not reflect differences in how consultation services are used in other provinces or countries. Thus, the advancement of knowledge could benefit from similar studies carried out in other contexts. It would also be important to survey community pharmacists in order to better understand their perceptions of the barriers and effects of their counselling activities.

Third, nonresponse bias cannot be measured in this study because we did not capture demographic information on the individuals who chose not to participate. Fourth, recall bias may be present, since participants were asked questions related to a previous consultation that was up to four weeks prior to the survey. Fifth, another limitation of our study is related to its cross-sectional nature. We thus encourage future studies to employ longitudinal approaches similar to Motulsky et al.^33^ Last, our data was collected before the COVID-19 pandemic. While the insights obtained with this dataset are especially valuable two years after the start of the current public health crisis, further research could verify if patients' experience with counselling services offered by community pharmacists have expanded.

## 5 Conclusions

This study highlights the central role played by community pharmacists in Quebec. Precisely, it reveals that the reasons for consulting a community pharmacist are diverse, most of which are related to medications and, to a lesser extent, health problems. Patients reported that community pharmacists were able to manage most of their consultations without referring them to other health care resources or professionals. Respondents also reported high levels of satisfaction with their consultations, especially adults aged 55+ and chronically ill patients.

## Consent for publication

Not applicable.

## Availability of data and materials

The dataset used and analyzed during the current study are available from the first author upon request.

## Transparency statement

The manuscript is an honest, accurate and transparent account of the study as it was conducted. No important aspects of the study have been omitted, and any discrepancies from the study as originally planned have been explained.

## Funding

This research project was funded by the *Association Québécoise des Pharmaciens Propriétaires* (AQPP). AQPP approved the design of the study and the questionnaire instrument.

## Declaration of Competing Interest

The authors declare that they have no competing interests.

## Figures and Tables

**Table:** 

Male
Female

**Table:** 

18–24 years
25–34 years
35–44 years
45–54 years
55–64 years
65 years and over
I prefer not to answer

**Table:** 

French
English
Other
English and French
French and other
English and other
Other and other
I prefer not to answer

**Table:** 

Elementary (7 years or less)
High school, general or vocational (8 to 12 years)
College (pre-university, technical training, certificate, accreditation or advanced diploma (13–15 years))
University certificates and diplomas
University Bachelor (including classical studies)
University Master's degree
University Doctorate (PhD)
I prefer not to answer

**Table:** 

For yourself:
For your child:
For a relative (ex. father, mother, brother, aunt):

**Table:** 

Yes
No

**Table:** 

Public insurance only (government – RAMQ)
Private insurance only
Public and private insurance
I am not sure

**Table:** 

None
Yes, one chronic disease
Yes, several chronic diseases

**Table:** 

In person at the pharmacy
By phone
Via a secure website

**Table:** 

Yourself
Your child
A relative (ex. father, mother, brother, aunt)

**Table:** 

Yes
No

**Table:** 

A family physician
A doctor or nurse at a walk-in clinic
A doctor at a hospital emergency room
A specialized physician
A nurse on the Info-santé phone line (811)
Another health professional (*specify*):

**Table:** 

Yes
No

**Table:** 

Take one or several new drugs or products available at the pharmacy
Stop taking one or several drugs or products available at the pharmacy
Adjust the dosage of a medication or the way to take a medication
Avoid taking a drug that is contraindicated for me, my child or my relative
Follow general health advice or action to be taken (e.g., hydrate well, limit salt intake, apply hot water compresses)
The pharmacist recommended none of the above

**Table:** 

A prescribed drug
Over the counter drug
A natural health product
Another product available at the pharmacy

**Table:** 

A prescribed drug
Over the counter drug
A natural health product
Another product available at the pharmacy

**Table:** 

A prescribed drug
Over the counter drug
A natural health product
Another product available at the pharmacy

**Table:** 

A prescribed drug
Over the counter drug
A natural health product
Another product available at the pharmacy

**Table:** 

Go to the emergency room
Go to a walk-in clinic
Schedule an appointment with a family physician
Schedule an appointment with a specialized physician
Schedule an appointment with another type of health professional
Call the Info-santé 811 phone line
To take another action (specify):
None of these actions were recommended by the pharmacist

**Table:** 

	Value
1 – Not satisfied at all	1
2	2
3	3
4	4
5 – Moderately satisfied	5
6	6
7	7
8	8
9	9
10 – Extremely satisfied	10

**Table:** 

Going to a hospital emergency room
Going to a walk-in clinic
Scheduling an appointment with a family doctor
Scheduling an appointment with a specialized physician
Scheduling an appointment with another type of health professional
Calling the Info-santé 811 phone line
Being absent from work or school
Did not avoid anything

**Table:** 

Recover more quickly
Avoid a worsening of health condition
Improve quality of life
Decrease anxiety level
Did not change anything

**Table:** 

Category	Reasons mentioned by participants for consulting a pharmacist
Medications	• Directions for medication use • Information about side effects of a medication • Information about non-prescription medicines • Eating and other restrictions associated with a medication • Reinforce medication information provided by family physician • Inquiry about possible drug interactions • Clarify physician recommendations with regard to medications
Health problems	• Questions related to… o Back pain o Stomach problems o Eye infection o Severe headache/migraine o Tooth pain o Insect bite o Skin problem o Hemorrhoid pain
Other issues	• How to use a medical device (eg, glucometer) and interpret data • Questions about reimbursement of prescription drugs • Questions about natural health products • Inform the pharmacist of insurance changes • Procedure for changing pharmacy • Reactivation of online portal account

## Appendix A Survey instrument

***Q1.*** *What is your gender?* ***(check one).***

*Q2. What is your age category?* ***(check one).***

***Q3.*** *What is the language you first learned at home in your childhood and that you still understand?* ***(check one).***

Q4. *What is the last year of education that you have completed?* ***(check one).***

***Q5.*** *How many times have you consulted with a pharmacist in the past 4 weeks?*

***Q6.*** *Do you currently have a family physician?* ***(check one).***

***Q7.*** *What type of drug insurance coverage do you have?* ***(check one).***

***Q8.****Have you been diagnosed with one or more chronic conditions (*e.g.*, diabetes, hypertension, arthritis, chronic pain, cancer, cardiac insufficiency, anxiety)?****(check one).***

*Please answer the following questions based on the past consultation you've had with a pharmacist that you consider to be the most important one.*

Q9. How did the consultation with the pharmacist take place? *(check one).*

***Q10.*** *What was the main reason for consulting with the pharmacist?*

Q10a. Who was this consultation for? *(check one).*

***Q11.*** *Before consulting with a pharmacist, did you try consulting with another health professional (walk-in clinic, emergency room, Info santé 811 phone line) for the reason you stated earlier?* ***(check one).***

***Q12. W*** *hat type of health professional did you consult with (or try to consult with)?* ***(check one).***

Q13. Did you actually see or talk to that person? *(check one).*

***Q14.*** *During the consultation, what did the pharmacist recommend? (check all the boxes that apply to your situation):*

***Q14a. For what type of drug or product exactly did the pharmacist recommend you to take one or several new drugs or products available at the pharmacy? (check one).***

***Q14b. For what type of drug or product exactly did the pharmacist recommend you to stop taking one or several drugs or products available at the pharmacy? (check one).***

***Q14c. For what type of drug or product exactly did the pharmacist recommend you to adjust the dosage of a medication or the way to take a medication? (check one).***

***Q14d. For what type of drug or product exactly did the pharmacist recommend you to avoid taking a drug that is contraindicated for me, my child or my relative? (check one).***

***Q15.****During the consultation, did the pharmacist recommend one of the following actions? (check all the boxes that apply to your situation)*.

***Q16.*** *On a scale from 1 to 10, what is your level of satisfaction with the consultation you had with the pharmacist?*

Q17. *The consultation with the pharmacist allowed me, my child or my relative to avoid: (check all the boxes that apply to your situation).*

***Q18.****The consultation with the pharmacist allowed me, my child or my relative to: (check all the boxes that apply to your situation)*.
